# Insights into the diversity and survival strategies of soil bacterial isolates from the Atacama Desert

**DOI:** 10.3389/fmicb.2024.1335989

**Published:** 2024-03-07

**Authors:** Alicyn Reverdy, Daniel Hathaway, Jessica Jha, Gabriel Michaels, Jeffrey Sullivan, Daniela Diaz McAdoo, Carlos Riquelme, Yunrong Chai, Veronica Godoy-Carter

**Affiliations:** ^1^Northeastern University, Boston, MA, United States; ^2^Facultad de Ciencias Básicas, Universidad de Antofagasta, Antofagasta, Chile

**Keywords:** extremophiles, microbial diversity, Atacama Desert, biofilm, antimicrobial production, antibiotic resistance, pigments

## Abstract

The Atacama Desert, the driest, with the highest radiation, and one of the most ancient deserts in the world, is a hostile environment for life. We have a collection of 74 unique bacterial isolates after cultivation and confirmation by 16S rRNA gene sequencing. Pigmentation, biofilm formation, antimicrobial production against *Escherichia coli* MG1655 and *Staphylococcus aureus* HG003, and antibiotic resistance were assessed on these isolates. We found that approximately a third of the colonies produced pigments, 80% of isolates formed biofilms, many isolates produce growth inhibiting activities against *E. coli* and/or *S. aureus*, and many were resistant to antibiotics. The functional characterization of these isolates gives us insight into the adaptive bacterial strategies in harsh environments and enables us to learn about their possible use in agriculture, healthcare, or biotechnology.

## Introduction

Extreme environments are challenging for life, yet bacteria have developed strategies for survival. One location in Chile characterized by its extreme environment is the Atacama Desert, located in northern Chile at the border with Bolivia and Argentina spanning about 128,000 km^2^. Bound by mountain ranges that prevent precipitation, the Atacama Desert is one of the most ancient and the driest temperate deserts in the world (Rundel and Villagra, 2007; Bull et al., 2018). Described as an extremobiosphere, the hyper-arid Atacama Desert contains a chain of Andean volcanoes, large salt flats, high mineral deposits, and Altiplano lakes (Rundel and Villagra, 2007; Rampelotto, 2013). Ranging between 2,000 and over 5,000 meters above sea level, the region experiences the highest levels of UV radiation in the world (Cordero et al., 2014, 2018) and low levels of oxygen, as well as large temperature fluctuations and extremely low precipitation levels (Rundel and Villagra, 2007; Bull et al., 2018; Cordero et al., 2018). Thus, the Atacama Desert is of increasing interest for understanding the microbial diversity and strategies to survive in extreme conditions including alkaline or acidic pH, temperature variabilities, water stress, and high UV radiation (Bull and Asenjo, 2013; Orellana et al., 2018), conditions that resemble those of other planets (Parro et al., 2011).

Extremophiles are bacteria that adjust to and survive in hostile environments once thought too harsh to sustain life (Rampelotto, 2013). These bacteria are categorized into two types: those that require the extreme condition, and those that tolerate extreme conditions, but can also grow optimally in “standard” conditions (Rampelotto, 2013). To thrive in harsh conditions, extremophiles use a variety of strategies to maintain their communities. These strategies have been extensively reviewed and include accumulation of certain molecules to counteract imbalances produced by the extreme conditions, production of specially designed enzymes and cell membranes, and enhanced DNA-repair mechanisms (Meyer, 2000; Gallardo et al., 2016; Órdenes-Aenishanslins et al., 2016; Narsing Rao et al., 2017; Mandakovic et al., 2020). Other such strategies include biofilm formation and production of inhibitory substances, antimicrobial activities, and pigment production (Hassan and Fridovich, 1980; Meyer, 2000; Hall-Stoodley et al., 2004; Azua-Bustos et al., 2012; Gallardo et al., 2016; Gerardin et al., 2016; Órdenes-Aenishanslins et al., 2016; Narsing Rao et al., 2017; Mandakovic et al., 2020). Biofilm is a community of surface attached bacterial cells encased in a self-produced, protective matrix that primarily consists of protein, exopolysaccharide, and extracellular DNA (Hall-Stoodley et al., 2004; Vlamakis et al., 2013). Biofilms enhance nutrient sharing, cell-cell communication (Hall-Stoodley et al., 2004; Vlamakis et al., 2013), and provide a protective barrier against antimicrobials, UV damage, and other pathogens (Hall-Stoodley et al., 2004; de Carvalho, 2017; Hall and Mah, 2017). As such, biofilm-producing bacteria are one of the leading causes of human and plant pathogenesis, consistent with its role as a bacterial survival strategy (Hall-Stoodley et al., 2004; Vlamakis et al., 2013).

Bacterial production of inhibitory substances is a competitive advantage since it allows the antimicrobial producing bacteria to fend off other colonizers and protect the population from further harm (Gerardin et al., 2016). For this reason, the search for novel antibiotics from environmental isolates is a very active area of research. The hope is to discover antibiotics to use against specific pathogenic bacteria without harming the general microbiome (Azua-Bustos and González-Silva, 2014; Lewis, 2017). Many groups have isolated and identified new compounds produced by *Actinobacteria* from the Atacama Desert, supporting the study of extremophiles for novel antibiotic discovery (Okoro et al., 2009; Rateb et al., 2011a; Santhanam et al., 2012a,b; Undabarrena et al., 2016; Goodfellow et al., 2018). But because bacteria are exposed to antimicrobials produced in their environment, they are apt to develop strategies for surviving these challenges (Ferri et al., 2017; Lewis and Caboni, 2017). Identifying naturally resistant bacteria will provide us in the future with mechanistic models to better understand how bacteria become antibiotic resistant as well as insights into how to combat this rapidly increasing problem. Indeed, antibiotic resistance has created a billion-dollar problem in the healthcare system (Ferri et al., 2017).

Microbial diversity studies from the Atacama Desert have only been published within the last 15 years (Bull et al., 2016). Overall, there is still a large gap in knowledge of the microbial diversity in the Atacama Desert. With better sequencing technology and increased characterization of cultivable isolates, we will gain a more complete view of what and how extremophiles live in their environment. Additionally, investigating which bacterial communities live in different locations is essential for understanding how they contribute to the environmental ecology and drive global dynamics.

Here, we provide a comprehensive picture of the microbial diversity and investigation into the survival strategies of bacteria living in the Atacama Desert. We sampled from 18 locations within the Atacama Desert. Further, we cultivated 74 unique isolates and performed characterization assays to identify pigment production, biofilm formation, production of inhibitory substances, and antibiotic resistance as probable survival mechanisms. In this study we provide insights into bacterial diversity and the strategies that bacteria use to survive in extreme environments.

## Results

### Locations and environmental sampling

**FIGURE 1:**
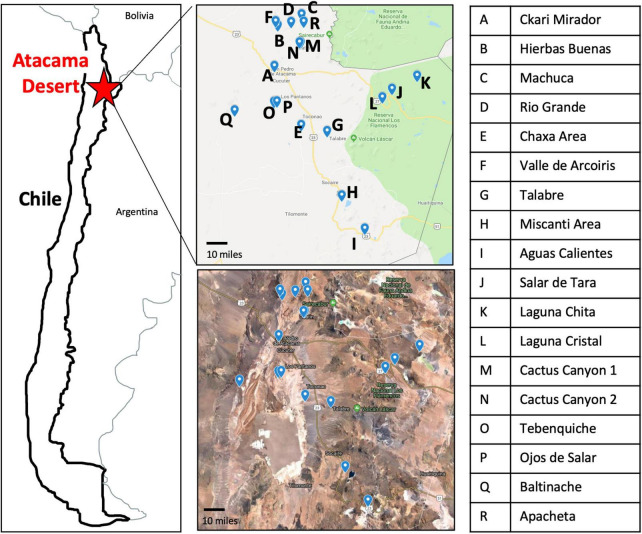
Map of the 18 sampling locations across the Atacama Desert. Sampling sites have clear geographical distinction as seen by Google Maps imaging. Letter key identifies the name of sampling location labeled on the maps.

The Atacama Desert exhibits the most extreme environment in Chile (Cordero et al., 2014, 2018; Bull et al., 2018). For this reason, we were curious to see what bacteria were present to identify what strategies they may use to survive in such a harsh environment. During the months of May and June of 2018, we traveled to 18 locations in the Atacama Desert (Figure 1 and Supplementary Figure 1). Each high-altitude location is distinct in its characteristics: fully barren to presence of low grasses and high to low mineral and salt content (Supplementary Table 1 and Supplementary Figure 1).

**FIGURE 2:**
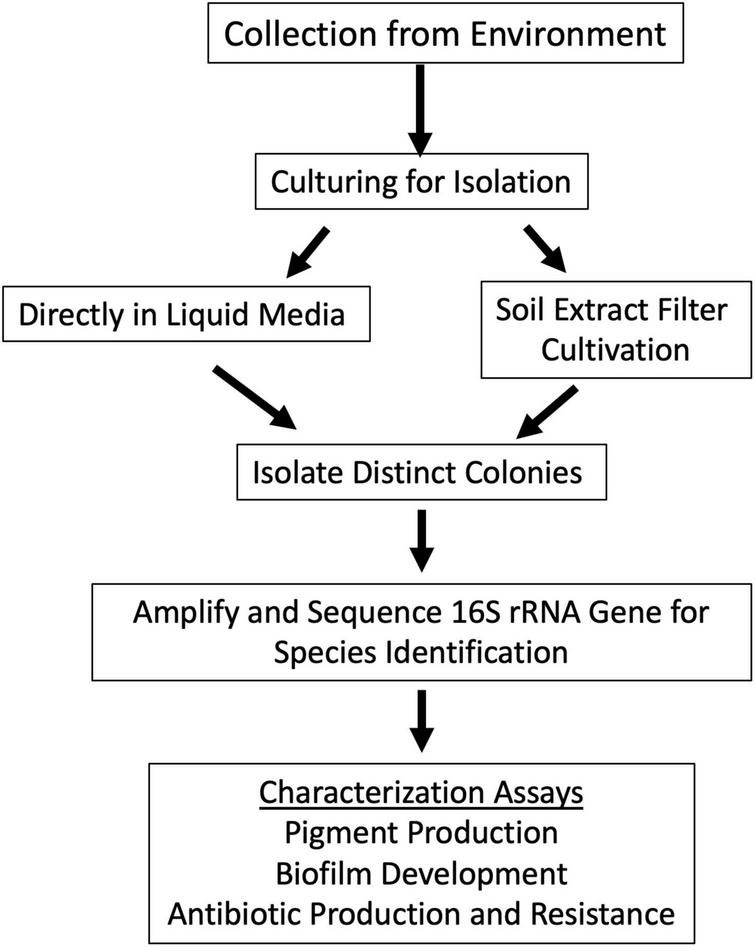
Schematic approach used for sample analysis and colony isolation. Samples were collected from locations in Figure 1. Samples were cultured to isolate individual bacterial species. This was done by two methods: inoculating the sample directly into culture medium or by scraping concentrated sample from soil extract filter disk and depositing it onto R2 agar plate. Bacterial colony growth was observed, and colonies were isolated until purity. Colony PCR of the 16S rRNA gene was used to identify closely related species of the colony isolates with different morphologies. Colony isolates were then assayed for pigment production, biofilm production, and antimicrobial production and resistance.

Specific sampling locations were chosen for their unique characteristics, high altitude, near water, barren, high salinity, etc. At each location, environmental soil samples between 1 and 5 cm were collected. The exact coordinates were recorded at each sampling location (Supplementary Table 1). Samples were then processed to cultivate bacterial isolates (Figure 2).

### Cultivation and isolation of unique isolates

Our next goal for gaining insights into the behavior of bacteria in the Atacama Desert was to cultivate and isolate single bacterial colonies from the samples that we collected. We found that, in general, isolates grew much better on the R2 medium than on the LB or 1:100 LB media plates. This was to be expected because bacteria from these locations are more accustomed to growing in low nutrient environments. We surmised that different colony morphology would be a good way as a first approximation to distinguish one isolate from the other. Therefore, isolates were further purified and examined on solid medium based on visual morphology differences.

**FIGURE 3:**
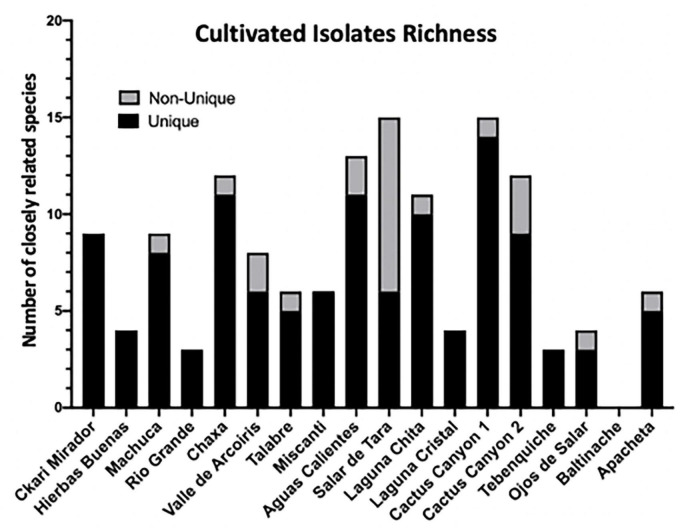
Among cultivable bacteria 74 unique colony species were isolated and identified. 142 distinct isolates based on colony morphology were isolated including 74 unique bacterial species. Isolates were cultured from environmental samples as described in Materials and methods. The strain identity of colony isolates with different morphologies were distinguished based on the 16S rRNA gene of each colony isolate amplified via PCR and sequenced. The 16S rRNA gene sequence was aligned to the NCBI database to identify colony genus, and the closest related species and/or strain using 97% identity as species separation and confidence. Distribution of isolates across sampling locations. Unique is defined as a bacterial isolates within the same location that has no other representative. Non-unique refers to the same closely related species found in different samples from the same location.

Results of the 16S rRNA gene sequencing identified 74 unique isolates out of the 142 purified colonies (52%) across all sample locations (Supplementary Table 2). The isolates belonged to 27 unique genera (19%) and closely related to 74 species (52%). Unique was defined as any isolate different to another within a given sample. For example, if two isolates were identified to be *Enterobacter* sp. from one location, but one was from a soil sample and another was from a moss sample, then they were considered unique. However, if two *Enterobacter* sp. isolates from a location were both from a soil sample and both had a percent identification above 97% by 16SrRNA, then they were considered sisters and non-unique. A distribution of the unique and non-unique isolates from the different sampling locations was made using the above definition (Figure 3).

Though 16S rRNA is not enough to identify species, 97% was used as the threshold of similarity that an isolate was closely related to a given species, though 98.65% has also been used (Yoon et al., 2017). In our case, any percent identity below 97% indicates that the isolate is likely a novel species, which we may be undercounting. Interestingly, 34% of our isolates had a percent identity below 97%, and 4% of all isolates had a percent identity below 90%. This suggests that we may have at least 48 new species within our collection (Supplementary Table 2).

Of all the culturable isolates, the most abundant genus was *Bacillus* sp. Forty-three isolates belonged to this genus and it was found in 13 locations (Supplementary Table 2). Bacteria closely related to *Bacillus simplex* were the most abundant among the enriched culturable species, and they were found in 10 locations (Supplementary Table 2 and Table 1). The next most abundant enriched culturable genus was *Arthrobacter*. Fourteen isolates (9.8% of all isolates) were found in 7 locations (Supplementary Table 2). These data could mean that *Bacillus* and *Arthrobacter* are non-discriminatory, can live in many diverse locations and are easily culturable.

**TABLE 1:** Cultivated isolates.

Closely related species	ID	Location	References
*Aeromonas aquatica* strain MX16A	98.00	Laguna Chita	Beaz-Hidalgo et al., 2015
*Agrobacterium rhizogenes* strain K599	89.57	Valle de Arcoiris	Valdes Franco et al., 2016
*Aeromonas* sp. AU20	93.19	Salar de Tara	Anda et al., 2015
*Bacillus atrophaeus SQA-17*	96.39, 99.05, 100	Ckari Mirador, Cactus Canyon 2, Laguna Chita	
*Bacillus atrophaeus* strain GQJK17	97.99, 98.03, 98.68	Valle del Arcoiris, Laguna Chita, Chaxa,	Ma et al., 2018
*Bacillus megaterium* strain JX285	100	Miscanti	Huang et al., 2019
*Bacillus mycoides* strain Gnyt1	99.71	Miscanti	Xiaomei and Yao, 2020
*Bacillus simplex* NBRC 15720 = DSM 1321	99.03, 98.19, 99.61, 99.13, 98.33, 88.70, 95.77 99.28, 94.67, 96.15, 98.61 98.49, 97.48, 99.01, 98.49, 98.31, 98.60, 95.69, 87.26, 94.37, 97.75, 99.81	Talabre, Cactus Canyon, Salar de Tara, Cactus Canyon 1, Laguna Chita, Rio Grande, Valle del Arcoiris, Aguas Calientes, Ckari Mirador, Miscanti	Gutierrez-Luna et al., 2020
*Bacillus* sp. X1(2014)	97.31	Miscanti	
*Bacillus subtilis* strain DKU_NT_03	99.99, 99.81, 95.90, 97.22, 96.50	Talabre, Aguas Calientes, Salar de Tara, Cactus Canyon 1, Laguna Cristal	Gutierrez-Luna et al., 2020
*Bacillus subtilis* strain SG6	97.20	Cactus Canyon 1	Zhao et al., 2014
*Bacillus thuringiensis* strain c25	97.93	Ojos de Salar	Shrestha et al., 2015
*Bacillus thuringiensis* strain SCG04-02	97.93	Ojos de Salar	Fu et al., 2017
*Brevundimonas* sp. LM2	96.79, 95.91	Chaxa, Cactus Canyon 1	Chen et al., 2020
*Enterobacter* sp.	98.93, 93.82, 98.54, 98.62, 98.53, 98.66, 99.90, 99.90	Cactus Canyon 1, Cactus Canyon 2, Tebenquiche, Laguna Cristal, Laguna Chita, Chaxa	Mafakheri et al., 2021
*Janthinobacterium agaricidamnosum* NBRC102515 = DSM 9628	96.58	Ckari Mirador	Graupner et al., 2015
*Microbacterium oryzae strain MB-10*	99.90	Cactus Canyon 2	Deb and Das, 2020
*Planococcus rifietoensis* strain M8	98.14, 97.01	Cactus Canyon 2, Chaxa	See-Too et al., 2016b
*Pseudomonas azotoformans* strain S4	97.15, 99.26	Tebenquiche, Rio Grande	Fang et al., 2016
*Pseudomonas brassicacearum* strain L13-6-12	99.70	Hierbas Buenas	Zachow et al., 2017
*Pseudomonas fluorescens* NCIMB 11764	87.03	Cactus Canyon 1	Silby et al., 2009
*Pseudomonas koreensis* strain D26	98.32	Tebenquiche	Lin et al., 2016
*Pseudomonas protegens* strain FDAARGOS_307	99.90	Cactus Canyon 1	Ramette et al., 2011
*Pseudomonas* sp. L10.10	97.40	Laguna Chita	See-Too et al., 2016a
*Pseudomonas* sp. M30-35	96.10	Laguna Chita	He et al., 2018
*Rahnella aquatilis* strain HX2	99.32	Machuca	Guo et al., 2012
*Serratia fonticola* strain GS2	96.52	Chaxa	Jung et al., 2017
*Serratia liquefaciens* strain FDAARGOS_125	99.80	Laguna Chita	Caneschi et al., 2019

Together, our data demonstrates success in bacterial cultivability from the extreme environments of the Atacama Desert. These isolates are representatives of where they came from and allow for the characterization of unknown species and identification of whether any of the known closely related species have unique characteristics because they were isolated from an extreme environment.

### Pigment producing isolates

**FIGURE 4:**
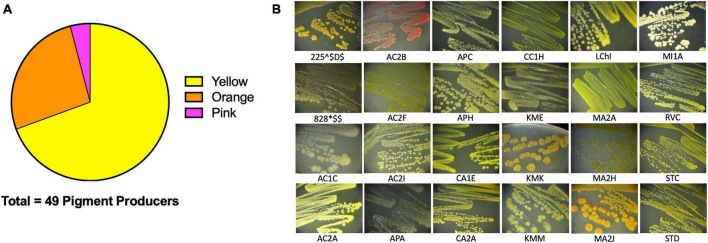
Extremophiles produce pigment. Pigment production was observed in colonies over 7 days. **(A)** 49/142 colony isolates produce pigments. **(B)** Sample images of pigmented colonies after 7 days of growth on R2 minimal medium agar at 25°C.

We then sought to investigate the capabilities of the Atacama Desert extremophiles we had cultivated, isolated, and closely identified by 16S rRNA. We surmised that by defining certain characteristics of these bacteria, we would gain insights into their strategies for survival in extreme environments. The first easily identifiable characteristic was that 34.5% of our isolates produced pigment (Figure 4A for quantification and Figure 4B representative isolates), most of which were cell bound. Of these isolates, 69.4% produced a yellow pigment, 26.5% produced an orange pigment, and 4.1% produced a pink pigment. Pigment production by bacteria is thought to be a protective mechanism against UV radiation (Azua-Bustos et al., 2012; Órdenes-Aenishanslins et al., 2016; de Carvalho, 2017; Narsing Rao et al., 2017). Many *Actinobacteria* and *Cyanobacteria* from salt flats and hyper-arid locations produce protective pigments (Bull and Asenjo, 2013; Narsing Rao et al., 2017). The Atacama Desert is known for its extreme levels of UV radiation (Demergasso et al., 2010; Cordero et al., 2018). It is plausible that these bacteria produce pigments to protect themselves from the DNA-damaging UV that they are exposed to while living in the Atacama Desert. We attempted to measure the UV resistance of some of our isolates, but it is a difficult endeavor since the pigments are secondary metabolites produced late in stationary phase. In fact, most of the isolates tested were UV-sensitive when growing exponentially compared to *Escherichia coli* (data not shown). Pigment isolation would allow determination of its functional role.

### Prominent biofilm formation is correlated to more extreme environments

**FIGURE 5:**
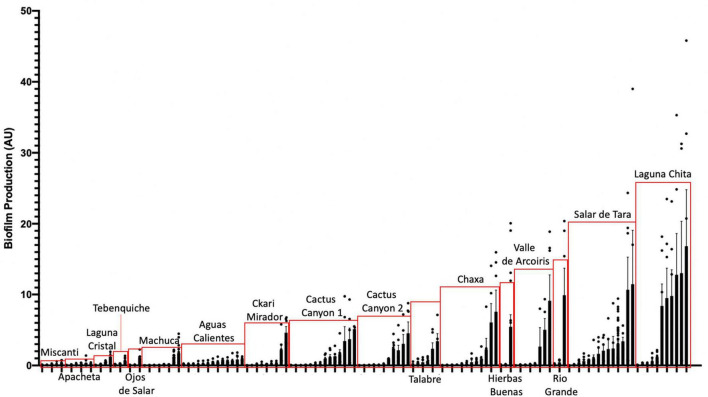
Extremophiles produce biofilm in more extreme environments. Colonies were assayed for biofilm formation by inoculating cells into R2 liquid medium in glass culture tubes. Cultures were grown statically at 25°C and quantified after 5 days of growth using the crystal violet (CV) staining assay (O’Toole, 2011). Biofilms were stained with 0.1% CV. Solubilized CV was then quantified using a spectrophotometer at OD_595_. OD_595_ was standardized by cell count (OD_600_) to quantify the amount of biofilm produced per cell (AU). Bars represent the average of two or more independent experiments for each individual isolate, dots represent biological replicates (*n* ≥ 6), and error bars represent standard deviation. Red boxes highlight isolates from the same location.

To assess whether biofilm formation was used as a survival strategy in the extremophiles of our collection, we performed biofilm assays. Briefly, cells were inoculated into R2 medium and incubated statically for 5 days at 25°C in glass tubes. Using a standard crystal violet (CV) assay as indicated in Materials and methods (O’Toole, 2011). Eighty percent of the142 isolates assayed under our conditions, attached to surfaces (Figure 5). This was determined by any value greater or equal to 0.05 AU, a value in which CV-stained cells were visible on the glass. The isolates closely related to *Bacillus simplex* exhibited the greatest biofilm production.

Also, of interest were the isolates that produced a pellicle or clumped biofilm. Isolates that produced a visible pellicle, a biofilm formed at the air-liquid interface (Vlamakis et al., 2013), were KMK, KMM, KME, APH, AC2F, and STB (Supplementary Figure 2). Additionally, KMK, RVC, AC2F, and APH produced a visible clumped biofilm while shaking. These are types of biofilms that might not be accurately quantified using the CV assay but are important examples of biofilm producers.

When comparing the amount of biofilm produced by isolates from the different locations, the ones from the most extreme environments were more likely to form biofilms. The bacteria inhabiting Laguna Chita had the most prominent biofilms; they attached to surfaces well. The location with the greatest number of biofilm formers was Salar de Tara (Figure 5). These locations are next to each other, are located at high elevation (> 4,200 meters above sea level), and are barren (Supplementary Figure 1 and Supplementary Table 1). The bacteria from these locations may use biofilm to facilitate nutrient sharing, protect against the high UV radiation, and enhance colonization. It is also of note that three isolates from Ckari Mirador produced pellicles/clumped biofilms. This location is exceptionally dry within the Atacama Desert and biofilm may be used by these isolates to enhance their survival in this extreme environment.

Together, our data indicate that biofilm production is a strategy that bacteria use to survive in the extreme environments found in the Atacama Desert.

### Extremophiles produce growth inhibitors against Gram-positive and Gram-negative bacteria

To identify isolates that produced growth inhibitors or antimicrobials, we performed a zone of inhibition assay against Gram-positive *Staphylococcus aureus* HG003 and Gram-negative *Escherichia coli* MG1655 as representatives of their respective groups. Isolates were grown shaking until late stationary phase and then spotted onto lawns of the target bacteria. Plates were incubated at 30°C, and the zones of inhibitions were measured after one and four days of growth.

**FIGURE 6:**
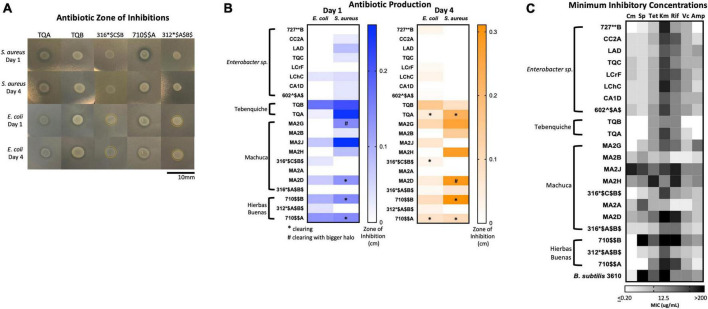
Extremophiles produce antimicrobials against *S. aureus* and *E. coli* and are naturally resistant to commercial antibiotics. **(A)** Images of select isolates demonstrating antimicrobial production. Clearing can be seen in isolates TQA, TQB, and 710$$A. Yellow circle delineates haloing. **(B)** Antimicrobial production assay demonstrated by a challenge assay in which a select set of isolates inhibit the growth of either *S. aureus* or *E. coli* or both. The zones of inhibition were measured after one and 4 days of incubation at 25°C. Stars indicate clearing and pound signs indicate clearing with a larger halo. Heatmap represents the average of three independent experiments each including three biological replicates (*n* = 9). **(C)** Minimum inhibition concentration (MIC) assay of select isolates demonstrated resistance to commercial antibiotics (Cm, chloramphenicol; Sp, spectinomycin; Tet, tetracycline; Km, kanamycin; Rif, rifampicin; Vc, vancomycin; Amp, ampicillin). In a 96-well plate, a select set of isolates were subjected to antibiotic challenge in 2-fold dilution in a range 200–0.2 μg/mL. Cells were inoculated into R2 medium and grown statically at room temperature for 3 days. Growth was quantified by plate reader at OD600. The MIC was determined as the lowest concentration at which cell growth was completely inhibited. Heatmap represents the average MIC across three independent experiments in singlet (*n* = 3).

There were two types of inhibition phenomena: haloing and clearing (Figure 6A). Haloing was when there was a visible ring of inhibition, however the target bacteria still grew. Most of the isolates demonstrated this phenomenon. Possible explanations for this haloing could be a result of inhibition then gained resistance by the target bacteria, or due to different growth rates where the test isolate grew after the target bacterium resulting in a delayed killing. Regardless, the haloing demonstrated an inhibition of the target bacterium by the test isolate. Further analysis of the resistant isolates would provide insights to any mechanism of resistance if the growth inhibiting compound were an antibiotic. The other phenomenon, clearing, suggests the produced antimicrobial is more potent. The isolates that demonstrated clearing are indicated by stars or pound signs (Figure 6A, B). Results show that many isolates produced growth inhibiting compounds against *S. aureus* and *E. coli* (Figure 6B). 16 isolates inhibited the growth of *S. aureus*, while 13 exhibited a challenge to *E. coli*. A loss of inhibition after 4 days showed that the target bacteria grew resistant to the challenge.

Of the isolates, TQA (closely related to *Pseudomonas koreensis*, ID: 98.32), TQB (closely related to *Pseudomonas azotoformans*, ID: 99.26%) and 710$AA (closely related to *Pseudomonas brassicacearum*, ID: 99.7) produced the most potent inhibitory compounds against both *S. aureus* and *E. coli* (Figure 6). This was seen by large clearing zones of inhibition (Figure 6A). Of more interest are isolates 316*$C$B$ (closely related to *Hafnia alvei*, ID: 98.1%), and 312*$A$B$ (closely related to *Hafnia alvei*, ID: 99.9%), both of which demonstrated killing against only the Gram-negative *E. coli* (Figure 6B). The identification of Gram-negative-specific antimicrobial compounds are of particular interest for drug discovery groups (Lewis, 2017).

Together, these data demonstrate that the chosen isolates produced growth inhibitory substances against *S. aureus* and *E. coli*. Antimicrobial production is an expected strategy that provides competitive advantage in an extreme environment.

### Extremophiles are naturally resistant to commercial antibiotics

Coming from remote environmental samples, none of these isolates have been exposed to commercially available antibiotics. Many of these isolates were also identified as genera that have developed multi-drug resistances (Supplementary Table 2; Santajit and Indrawattana, 2016). Such examples include *Enterobacter* sp., *Acinetobacter* sp., and *Pseudomonas* sp (Supplementary Table 2). We were curious as to whether any of the isolates were inherently resistant to commercial antibiotics; thus, we performed a minimum inhibitory concentration (MIC) assay using antibiotics with different mechanisms of action. The MIC was measured for the same 21 isolates from Supplementary Table 3 used in the antimicrobial production assay. We determined the MIC for ampicillin (targets cell wall), vancomycin (targets cell wall), rifampicin (targets RNA polymerase), kanamycin (targets 30S ribosomal subunit), tetracycline (targets 30S ribosomal subunit), spectinomycin (targets 30S ribosomal subunit), and chloramphenicol (targets 50S ribosomal subunit) at a range of 200 to 0.2 μg/mL (Lewis, 2013). The MIC was defined as the lowest concentration of antibiotic that completely inhibited growth.

Results demonstrated that many isolates had high MICs to multiple antibiotics (Figure 6C and Supplementary Table 4). High MIC suggested resistance while low MIC suggested susceptibility to a particular antibiotic. Overall, the isolates had the highest MICs to ampicillin and the lowest MICs to kanamycin (Figure 6C and Supplementary Table 4). The isolates with the highest MICs were TQB (closely related to *Pseudomonas azotoformans*: 99.26%) and MA2B (*Microbacterium* sp. ID: 99.2%) (Figure 6C and Supplementary Table 4). For TQB, its lowest MIC was 6.25 μg/mL against kanamycin and tetracycline (Supplementary Table 4). TQB also had growth at 200 μg/mL antibiotic concentration for four antibiotics (ampicillin, vancomycin, spectinomycin, and chloramphenicol) indicating it was not susceptible to these antibiotics at all (Supplementary Table 4). For MA2B, its lowest MIC was 12.5 μg/mL against vancomycin (Supplementary Table 4). The most susceptible isolate was MA2J (closely related to *Exiguobacterium antarcticum* B7 ID: 98.7%) (Figure 6C and Supplementary Table 4). Its highest MIC was 6.25 μg/mL against spectinomycin and chloramphenicol (Supplementary Table 4). What gives us confidence for this assay is that isolates from the same genera had the same MIC patterning (Figure 6C). Examples include the *Enterobacter* sp. isolates (727**B*, CC2A, LAD, TQC, LCrF, LChC, CA1D, and 602^$A$) and the *Pseudomonas* sp. isolates (TQA, TQB, and 710$$A).

Together, these data demonstrate that isolates from remote extreme environments with little to no encounter with commercial antibiotics, have multidrug resistances.

## Discussion

In this study, we provide insights into the diversity and functionality of culturable environmental isolates from Atacama Desert (Figures 1–4 and Supplementary Figures 1, 2). We identified the dominant phyla to be *Actinobacteria* and *Proteobacteria* in Atacama soils (Supplementary Table 2). A culture-dependent strategy and cultivated bacteria from the collected samples led to the isolation of 74/142 unique species (Supplementary Table 2 and Table 1).

Before this study, no one as far as we could find had reported the presence of bacteria in any of our locations except for Chaxa, Tebenquiche, and Miscanti (Demergasso et al., 2004, 2008; Dorador et al., 2018). These studies, however, were on water samples. Here, we add to the collection of data that others have provided to increase the understanding of the microbial diversity in new locations within the Atacama Desert.

The isolates that we cultivated must be extremotolerant bacteria; those that can tolerate extreme conditions but can also successfully grow under “normal” physiological conditions (Rampelotto, 2013). Being that we cultivated under the latter conditions, it is highly probable that other bacteria can be cultivated from these samples using more selective conditions.

Many previous studies lacked research on bacteria physiology (Demergasso et al., 2004, 2008, 2010; Drees et al., 2006; Dorador et al., 2010, 2018; Bull et al., 2016; Mandakovic et al., 2018a,b; Schulze-Makuch et al., 2018; Uritskiy et al., 2019). Being that a third of our isolates produced a pigment, there is strong likelihood that it is made for protection in its environment (Figure 4). One example of pigments used for survival is the production of pyocyanin and pyoverdine from *Pseudomonas* sp. Pyocyanin is a blue-green pigment produced by *Pseudomonas* sp. that acts an antimicrobial, but also has been shown to have siderophore activity to uptake environmental iron (Hassan and Fridovich, 1980; Jayaseelan et al., 2014). Pyoverdine, is a yellow-green pigment and siderophore secreted by *Pseudomonas* sp. during iron starvation as a way to sequester iron(III) (Meyer, 2000). Indeed, the identified *Pseudomonas* genera isolates were seen to produce a secreted yellow pigment as exemplified by TQA, TQB, RGA, APA, and 710$$A (data not shown). Other groups have also seen that *Actinobacteria* and *Cyanobacteria* from the Atacama Desert produce melanins, scytonemin, carotenoids, chlorophylls, and other pigments (Azua-Bustos et al., 2012; Bull and Asenjo, 2013).

To date, no one has shown biofilm production data of microbial isolates from the Atacama Desert. 80% of our isolates produced a biofilm and there were many distinct types of biofilms produced (Figure 5 and Supplementary Figure 2). The bacteria from these locations may use biofilm as a survival strategy to facilitate nutrient sharing, protect against the high UV index, and enhance colonization. The two locations of high biofilm production, Laguna Chita and Salar de Tara, were devoid of plant life. The presence of biofilm-producing bacteria may also provide plant-promoting effects to the few low grasses that do live in the area.

Our study showed that some isolates produce antimicrobials against Gram-positive *S. aureus* and Gram-negative *E. coli* (Figure 6). This was not surprising because the most dominant phylum was *Actinobacteria* (35.9%) and because many other groups have been working with these antibiotic producers (Okoro et al., 2009; Rateb et al., 2011a,b; Santhanam et al., 2012a,b; Undabarrena et al., 2016; Goodfellow et al., 2018).

Lastly, we found that many isolates had high MICs against commercially produced antibiotics indicating inherent antibiotic resistance (Figure 6C). Being from remote environments, these isolates have theoretically not encounter commercial antibiotics until now. Because they are resistant to them, they must have intrinsic strategies to resist the antibiotic challenge. We believe this resistance has developed by three possible means: 1. they face antibiotic challenge in their environments, 2. mutagenesis because of being subjected to high levels of UV radiation, and 3. they are extremophiles and have strategies to withstand exogenous stress.

Many commercially available antibiotics were originally discovered to be produced by soil bacteria. Vancomycin, for example, was discovered from soil isolate *Streptomyces orientalis* (Levine, 2006). It is, therefore, not surprising that our isolates had relatively high MICs to this antibiotic (Figure 6C and Supplementary Table 4) since they are isolated form the soil. Environmental bacteria use antimicrobial production as a competitive advantage, and they must develop ways to resist these challenges and outcompete the bacteria producing them. Our isolates were most likely exposed to similar type of antimicrobials in the soil and therefore, have developed resistance mechanisms that enable their growth when challenged with commercial antibiotics (Hibbing et al., 2010).

Mutagenesis is the result of a variety of genomic insults coming from diverse sources including reactive oxygen species, methylating agents, or ionizing radiation (Chatterjee and Walker, 2017). UV radiation is one of the most damaging abiotic factors to the cell that directly damages the DNA by formation of thymidine dimers (Chatterjee and Walker, 2017; Cordero et al., 2018). As a result, mutations occur, which could be favorable to the survival of bacterial species. Antibiotic resistance is one advantage that can be gained by mutagenesis (Li et al., 2019). Therefore, the more UV that the bacteria are exposed to, the higher the probability that they will accumulate mutations to confer antibiotic resistance.

The third explanation for higher resistance is that these extremophiles inherently have strategies to survive stress. These bacteria may have improved their cell membranes, enzymes, DNA repair machineries, among other adaptations that allow them to resist chemical challenges in addition to the condition they already survive (Hibbing et al., 2010; Orellana et al., 2018). Efflux pumps, for example, have been found to increase antibiotic resistance and tolerance and are also found in many high metal tolerant extremophiles (Lewis, 2017; Orellana et al., 2018). Such examples include *Arthrobacter* sp., and *Flavobacterium* sp., which were genera isolated and used in the MIC assay (Supplementary Table 4 and Figure 6C; Orellana et al., 2018). Studies have also shown that extremophiles from the Atacama Desert have high resistance to arsenic due to the arsenic-rich Atacama brooks and soils (Escalante et al., 2009; Azua-Bustos et al., 2012). Examples from our isolate list include *Pantoea*, *Serratia*, *Hafnia*, *Microbacterium*, *Exiguobacterium*, and *Pseudomonas* (Supplementary Tables 2; Orellana et al., 2018). Indeed, our most resistant isolates were TQB (closely related to *Pseudomonas azotoformans*: 99.26%) and MA2B (*Microbacterium* sp. ID: 99.2%) (Figure 6C). Their resistance mechanisms for high metals and arsenic toxicity may lead to their naturally occurring antibiotic resistance.

## Conclusion

There are many reviews covering the advantages of extremophiles, especially in the light of bioremediation (Azua-Bustos and González-Silva, 2014; Bull et al., 2016; Orellana et al., 2018). It is important to go beyond microbial diversity analyses and isolate individual bacteria to learn what they are really doing. Cultivation is obviously a challenge but learning from extremophiles will allow us to understand the adaptation mechanisms that microbes use to survive in their environments. Their more sophisticated machineries will give insight into the metabolic and synthesis pathways that their non-extreme and pathogenic relatives utilize. This will lead to discoveries of new enzymes, pigments, and compounds that can be used in biotechnology, agriculture, and healthcare.

## Materials and methods

### Sample preparation and storage

Environmental samples were collected during the months of May and June of 2018. At each location soils were sampled from in between 1 and 5 cm and collected into sterile 2 mL microcentrifuge tubes using sterile tools. The date and exact coordinates were recorded at each sampling location (Supplementary Table 1). It is important to note that samples from Chaxa, Miscanti, Aguas Calientes, and Salar de Tara were taken from outside of the national parks. Samples, e.g., soil, water, moss, etc. were maintained at room temperature while in the field. Once brought to the laboratory, 20% (v/v) glycerol in water was added and stored at −80°C.

### Bacteria isolate cultivation

Bacterial cultivation was performed using two approaches: direct cultivation and soil extract filter cultivation (Figure 2). For direct cultivation, ∼100 mg of soil was added directly to liquid cultivation medium. Four media were used: Luria Benton (LB) liquid and solid agar, 1:100 diluted LB liquid and solid agar, R2 liquid and solid agar (BD Difco, Franklin Lakes, NJ, USA). Liquid cultures and plates were incubated at room temperature overnight. Liquid cultures that had visible growth, as seen by turbidity, were deposited onto the surface of solid agar plates of the same medium for colony formation. All cultures and plates were grown statically at room temperature.

The vacuum filter disks were collected to trap and concentrate any bacteria that were in the soil onto the filter paper. A sterile stick was used to scrape the filter disk and was resuspended into 100 μL of sterile water. The resuspension was then plated onto R2 medium agar plates. Plates were incubated at room temperature for colony formation.

Colonies were struck onto R2 solid agar plates until single distinct colonies were observed. After obtaining all our isolates, potential sisters were removed by the following steps. First, colonies of the same morphology from the same location were removed. Second, colonies of the same morphology across the entire isolate collection were removed. At this point, isolates were stocked in 20% (v/v) glycerol in water and stored at −80°C.

For all subsequent cultivation and assays, R2 solid agar and liquid medium were used and incubated at 25°C. Isolates were observed to grow well in these conditions. Isolates were named according to a numbering system based on location, sample number, and medium type.

### Species identification

To identify the species of each cultivated isolate we amplified and sequenced the 16S rRNA gene from the bacterial chromosome. A single colony was resuspended in 20 μL of water and then used as the template in the polymerase chain reaction (PCR) reaction. The 16S rRNA gene was amplified using OneTaq Polymerase Master Mix (Fisher Scientific, Agawam, MA) and bacteria specific 16S rRNA gene primers 8F (5′-AGAGTTTGATCCTGGCTCAG-3′) and 1492R (5′-GGTTACCTTGTTACGACTT -3′) (Galkiewicz and Kellogg, 2008). For those isolates that colony PCR was not successful, genomic DNA was extracted using chemical extraction and used as the template for PCR amplification (Sambrook and Josehp, 2001). The PCR products were sent for purification and Sanger Sequencing service at Eurofins Genomics (Louisville, KY, USA). Sequences were identified using NCBI Nucleotide Blast alignment.

We chose a 97% percent identity to determine the more closely related species. If the identity is lower than 97%, the isolate is considered a different and/or unidentified species. To identify unique isolates, we defined a sister isolate as any isolate similar in colony morphology to another isolate within a given sample. This was confirmed by 16S rRNA sequencing. If the percent identity was not available, then both isolates were kept. If both percent identities were above 97%, the isolate with the lower percent identity was removed.

### Pigment identification

Pigment production was observed by tracking morphology over the course of 7 days. Samples were categorized into yellow, orange, and pink pigment production. Results were reported as a percentage of all the isolates that produced a pigment.

### Biofilm production assay

Isolates were grown overnight in R2 liquid medium shaking at 25°C. Biofilm assay cultures were then inoculated 1:1,000 into R2 liquid medium in small glass culture tubes and grown statically at room temperature. All strains were tested for biofilms after 5 days of static growth. At harvest, the OD600 was measured by spectrophotometer to quantify cell density. Quantification of biofilm production was done by adaptation of published protocols (O’Toole, 2011). Briefly, cultures were decanted and rinsed with phosphate buffer saline (PBS). Any leftover liquid was pipetted out, and the tubes dried for 10 min in a fume hood. Each tube was filled with 0.1% (w/v) crystal violet stain and incubated for 15 min at room temperature. The stain was decanted, and the tubes were twice washed with PBS. Excess liquid was pipetted out and dried for 10 min under a flow hood. The remaining dye adhered to the biofilm was then dissolved in 30% (v/v) acetic acid in water for 15 min. This solution solubilized the dye. The crystal violet was measured at OD_595_. All experiments were performed in biological triplicates. R2 liquid medium was used as a blank control and the isolate 225*$C$, a known-biofilm producer, was used as a positive control in each experiment. For quantification of biofilm production, all OD_595_ values were subtracted by the blank control OD595 value. This new value was then divided by the isolate’s respective OD600 value to standardize all the cultures by cell count. Outliers were removed by Grubbs outlier test. Replicates were averaged, and the standard deviation of the mean was calculated to evaluate error.

### Antimicrobial production assay

Test isolates were sub-cultured 1:100 from saturated cultures into R2 liquid medium in triplicate glass tubes. The cultures grew shaking at 25°C for 20 hrs. Lawns of *S. aureus* HG003 and *E. coli* MG1655 were prepared by sub-culturing cells 1:100 from saturated cultures into LB liquid medium. Bacterial cells were grown shaking at 30°C for 1 h until reaching about OD_600_ = 0.1, which were then diluted to an OD_600_ = 0.02 in R2 liquid medium. The diluted cells were used to flood R2 solid agar plates and further dried in a flow hood for 30 min. 3 μL of the environmental isolates’ saturated cultures were spotted onto the *E. coli* and *S. aureus* lawns. For comparison purposes, ampicillin (25 mg/mL) was spotted as a positive control since the strains are ampicillin susceptible, and R2 liquid medium was spotted as a negative control. Plates were incubated at 25°C. After one and four days of incubation, the zones of inhibition were measured from the edge of the grown colony to the edge of the inhibition zone. Three independent experiments each with biological triplicates were performed. The average zone of inhibition was calculated for all three experiments (*n* = 9).

### Minimum inhibitory concentration (MIC) dilution assay

Selected isolates were challenged against commercial antibiotics ampicillin, rifampicin, vancomycin, kanamycin, tetracycline, spectinomycin, and chloramphenicol (all purchased from Fisher Scientific, Agawam, MA, USA or Sigma Millipore, St. Louis, MO, USA) in 96-well plates. Media was supplemented with antibiotic in a 2-fold dilution ranging from 200 to 0.2 μg/mL final concentration. Rifampicin was diluted 2-fold in a range 164 μg/mL to 0.2 μg/mL final concentration. Cells were inoculated at a 1:100 dilution from a saturated culture. Wells with no antibiotic, no cells, and R2 supplemented with antibiotic vehicle were included as positive, negative, and vehicle effect controls. *Bacillus subtilis* NCBI 3610 was used as a control strain with known MICs to these antibiotics. Assay plates were covered with a sealing film and incubated at room temperature for 3 days. After 3 days, isolate growth was quantified using a plate reader at OD600. The MIC was determined as the concentration that fully inhibited growth. The experiment was performed three independent times each in biological singlet. The MIC was calculated to be the average from the three experiments.

## Data availability statement

The original contributions presented in the study are included in the article/Supplementary material, further inquiries can be directed to the corresponding author.

## Author contributions

AR: Conceptualization, Investigation, Methodology, Writing – original draft. DH: Investigation, Writing – original draft. JJ: Investigation, Writing – original draft. GM: Investigation, Writing – original draft. JS: Investigation, Writing – original draft. DM: Writing – original draft. CR: Methodology, Resources, Writing – original draft. YC: Investigation, Resources, Supervision, Writing – original draft. VG-C: Conceptualization, Data curation, Formal Analysis, Funding acquisition, Investigation, Methodology, Project administration, Resources, Supervision, Validation, Writing – original draft, Writing – review & editing.

## Supplementary material

Supplementary Figure 1 Images of sampling locations. Images taken at the time of sampling in each location. The sample place name is indicated.

Supplementary Figure 2 Images of unique biofilm producers. Side view images taken of unique biofilm producers. Pellicle biofilms were produced after 5 days of static incubation at 25°C in R2 liquid medium. Shaking biofilms were observed as excess matrix and cell clumping. Cells were grown overnight shaking at 25°C in R2 liquid medium. Crystal violet-stained biofilms were imaged after 5 days of static incubation at 25°C in R2 liquid medium.
