# Supplementary figures and images for: Insights into the diversity and survival strategies of soil bacterial isolates from the Atacama Desert
Source: Front Microbiol. 2024 Mar 7;15:1335989. doi: 10.3389/fmicb.2024.1335989 (PMC10955380; doi:10.3389/fmicb.2024.1335989)

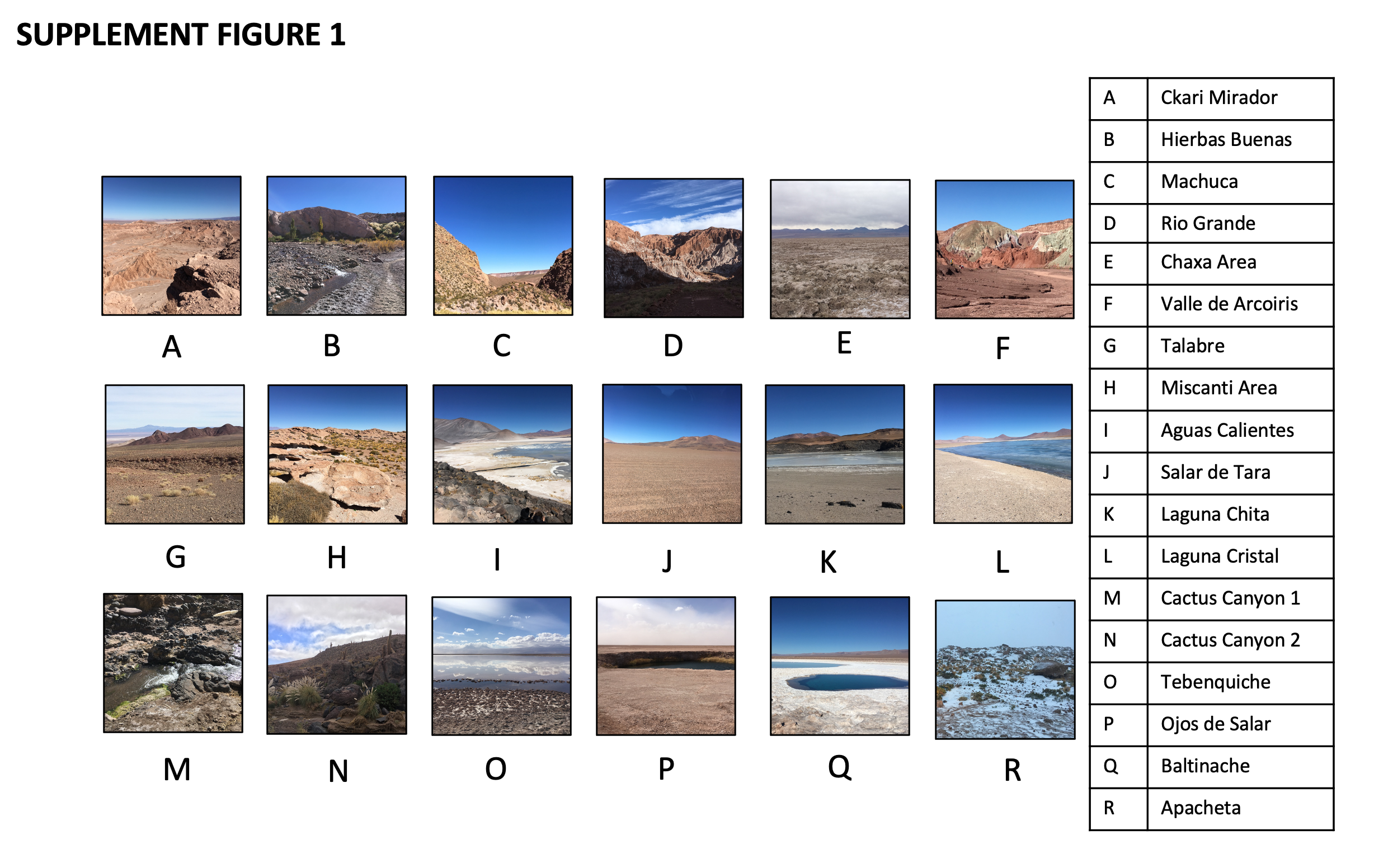

Supplement: Supplementary Figure 1 — Images of sampling locations. Images taken at the time of sampling in each location. The sample place name is indicated. [file Image_1.TIFF]

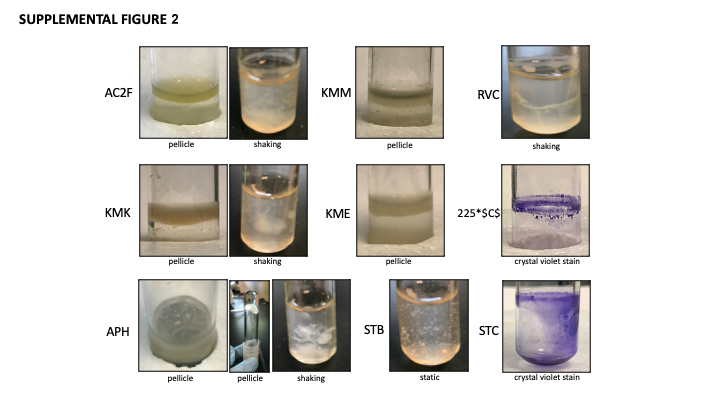

Supplement: Supplementary Figure 2 — Images of unique biofilm producers. Side view images taken of unique biofilm producers. Pellicle biofilms were produced after 5 days of static incubation at 25°C in R2 liquid medium. Shaking biofilms were observed as excess matrix and cell clumping. Cells were grown overnight shaking at 25°C in R2 liquid medium. Crystal violet-stained biofilms were imaged after 5 days of static incubation at 25°C in R2 liquid medium. [file Image_2.tif]
